# It's Elementary: Science Buddies Bring Biology to Life

**DOI:** 10.1371/journal.pbio.1000182

**Published:** 2009-08-25

**Authors:** Rachel D. Fink

**Affiliations:** Department of Biological Sciences, Mount Holyoke College, South Hadley, Massachusetts, United States of America

As a developmental biologist with a passion for teaching, I enjoy explaining my work on cell rearrangements in the yolk sacs of killifish embryos to audiences ranging from first-year undergraduates to professional colleagues. Becoming a parent offered new opportunities. When my daughter entered our local elementary school, I wanted to find a way to share my professional life with her teachers. My modest desire to lend a helping hand evolved into a “Science Buddies” program, where undergraduate science majors are matched with elementary classrooms. Conceived as a way to help enrich the local science curriculum, the program has served as a training ground for young science educators, opening the eyes of college students to the joys and challenges of primary school teaching.

Volunteering in the school started with an offer to bring in animals from my marine aquaria. I packed up a “Tupperware tidepool,” spread newspaper on the second-grade classroom floor, and passed around sea urchins and horseshoe crabs. The kids were most interested in the basics—Are they poisonous? Where did I get them? Do horseshoe crabs taste good? My visits soon expanded beyond my daughter's class, as other teachers stopped me in the halls or sent e-mails, asking if I could bring something to their classrooms, too. One hardy sea cucumber entertained dozens of seven-year-olds and forged a link that brought my expertise to new audiences.

Once I had established myself as a local scientist who loved being in the school, I was asked to be the parent liaison for a program that supplies elementary classrooms with chilled aquaria and salmon embryos for the students to hatch and rear. After four months of stewardship, the larvae are released in watershed habitats as part of an attempt to reestablish local populations. I showed time-lapse video sequences of early fish development to the fourth-grade classes, explained how a spherical egg is transformed into a small fish-on-a-ball-of-yolk, and led discussions about the salmon life cycle.

When the elementary school purchased a dissecting microscope, video camera, and monitor, the microscopy cart mostly sat in the supply closet gathering dust—not because the teachers lacked the interest in the new equipment, but because they lacked the time. During a sabbatical, I made myself available to the teachers as a microscopy tech, bringing the setup into classrooms to look at whatever the class was studying—caterpillars, rocks, flower parts, etc. This was an excellent way to meet other teachers and be introduced to the science curriculum in the different grades. We expanded the salmon project by looking at the larvae under the microscope, and I brought in zebrafish embryos so the students could see much earlier stages of fish development. The first time the students saw a beating heart and blood flowing through the yolk sac of the transparent zebrafish embryos they were awestruck. They looked at the veins on the inner side of their wrists, felt their own heartbeat, and made connections between what they saw under the microscope to their own circulatory system.

To ensure that these hands-on lessons would continue after my sabbatical ended, I started Science Buddies in 2005. I asked for volunteers among my undergraduate students and had them fill out a written application, looking for talented students who also had experience working with children. As Buddies, they made the commitment to weekly class visits, taking resources from the college, and to send me e-mail reports detailing what they did. I was awarded a US$1,000 “innovation grant” from my college to support this project, and the funds allowed us to purchase materials for the classroom teachers. The program started slowly, with four undergraduates in two different classrooms acting simply as “extra pairs of hands,” as students and teachers learned to work together.

Armed with bouquets of flowers for dissection, magnets, and an inflatable solar system purchased as part of the Buddy program, teachers found it easier to bring new depth to their science classes. When a third-grade class read a book featuring an owl as one of the main characters, we tied their reading unit to the dissection of owl pellets in science class. A third-grade teacher asked if we could help bring a unit on germs to life by providing some materials, so I taught Buddies to pour agar plates and stocked the library with many copies of a children's book of bacteriology. Over the next couple of weeks, the class swabbed surfaces in their classroom to see what would grow, and the students were amazed that water fountains and desktops provided just as many bacteria and fungi as the bottom of someone's shoe or a dirty corner of the floor. The Buddies brought a video cart into class to look at the results, and a lesson that had once consisted of a rather mundane worksheet became a lively “germfest.” This lesson spread from one class to the other third-grade classes, and now, during certain weeks of the year, you can see kids wandering the halls, Q-tips in hand. For the unit on “Animals of Massachusetts,” each child chooses an animal to study. One Buddy worked with a very shy boy who had little enthusiasm for the assignment until she lent him a coyote skull from one of the college's display cases. He was transformed by this and gave a dynamic report that had his classmates begging to touch the skull.

The curriculum at the local elementary school does not correspond to the departments found in a liberal arts college; “science” encompasses weather, butterfly life cycles, magnetism, and the solar system. Because I am a biologist, I chose undergraduates on the basis of their talents in biology courses. When asked to come up with experiments on magnetism, some of the Buddies had to go back to high school notebooks for inspiration or talk to friends who were majoring in other fields. They found websites devoted to elementary education, chose an experiment on iron in breakfast cereal, borrowed some strong magnets from the college physics department, and added something completely new to the curriculum. This stretched the Buddies in ways they had not been asked to do before and helped them make connections between their different college science classes. Indeed, a recent e-mail from the student in charge of the Mount Holyoke Physics Club asking to participate in the Buddies program is evidence of the cross-disciplinary attraction of working in elementary classrooms, and having physicists working with the schoolchildren will greatly enrich the program.

As the Science Buddies became trusted partners in the classrooms, the teachers asked for our help with the fourth-grade science fair. This had been a regimented event requiring every child to do exactly three replicates of their experiments, based on old, poorly photocopied handouts titled “The Scientific Method.” It was hard to find judges, and many students didn't really understand what was going on. One teacher confessed that she hated the science fair because it was so competitive. “Each of my students marched into the Fair an enthusiastic scientist, ready to show his or her poster,” she said. “Every child but one left the fair a loser.” After a brainstorming session, the teachers decided to get rid of the competition (all participants would be acknowledged, without naming a winner) and to help their students plan experiments in more individualized ways. The fourth graders wrote up their preliminary ideas for projects, and the Buddies and I joined the teachers to meet with every group, sharing ideas about how to ask questions, what specific controls might be included, what the data might look like, and how to present results. The process of taking a child's enthusiasm and finding a scientific question was both challenging and rewarding for the Buddies. In one case, two girls wanted to “grow seeds in different kinds of soda.” With prodding, the young students were able to articulate an interest in watering plants with different kinds of liquids and come up with valid predictions about how the plants might do—“We think the ones watered with soda will do better because plants need sugar to live, and soda has lots of sugar.” Another pair of children wanted to X-ray the jaws of their pets to see which set of teeth (dog or cat) were better. After much consultation, they designed an experiment to see which animal could eat more biscuits in a certain amount of time. While this project had some major design flaws, the children learned how to ask a question, collect data, perform replicates, draw graphs, and even included X-rays of dog and cat skulls they found on the internet. The evening of the Science Fair has become a lively event, scheduled to allow for participation of the Buddies, who meet the parents of “their” children, comment on the projects, and give out ribbons to all participants.

As the program grew, coordination became more important to match teachers with specific needs with appropriate Buddies. Some teachers were very confident teaching science and were terrific matches for any undergraduate. Other teachers wanted a very strong student to be in charge of the lessons, trusting that I would help oversee the quality of the material presented. This year we had ten undergraduates working in eight different classes, with a wide range of teacher–Buddy models.

I started out thinking that the Buddy program would be a way to aid the elementary school teachers and hadn't really considered what effect it might have on the college undergraduates. I now realize that this project, and others like it, can serve as a training ground for science educators by inspiring undergraduates to translate their enthusiasm for science into a powerful teaching tool. Among our undergraduate biology majors are those who have a clear goal in mind—they want to be doctors, veterinarians, college biology professors, or laboratory researchers. Past experiences, such as summers spent shadowing a physician or working in a lab, have helped them focus on a career path. Science Buddies, mentored by the classroom teachers and supported by college professors, are able to experience elementary education in a supervised, low-stress, highly creative way. They quickly realize that memorizing the steps of the photosynthetic pathways may get them an “A” in Intro Bio, but may not be useful when talking with eight-year-olds. If the Buddy is able to make the leap from student to teacher—if she can take all she has learned about plants in her college courses and bring a wilted lily to life for a group of third graders—she may find a sense of satisfaction and achievement that will stimulate her to choose education as her life's work. Indeed, a few Buddies have gone on to get their teaching licenses, and others are in graduate programs in science education.

The Buddy program evolved in a unique and quirky way, based on the specific teachers, classroom dynamics, and energy levels of all involved—not having a firm plan was a key to its success. It is the classroom teachers who are the experts; I did not come into the school with a prescription or recipe, and the Science Buddy program does not set the elementary science curriculum. As a scientist, it was important for me to listen to the teachers and help catalyze the changes they wanted to make. Public schools around the country have science fairs, astronomy nights, organic vegetable gardens, and field trips to local science museums—all opportunities for professional scientists to become involved with already-existing endeavors.

In my case, the local elementary school principal welcomed outside volunteers and supported innovation. There was no requirement for any class to work with a Buddy, and the self-selected group of teachers who participate have proven to be talented mentors. The undergraduates are more than willing to do this as volunteer work or for academic credit, finding their classroom visits a welcome break from their regular collegiate responsibilities, and most have been inspired to learn more about teaching as a profession. A small budget empowers teachers and Buddies to come up with creative ideas. One hopes that many colleges and universities could find modest funds to support this kind of community-based learning for their undergraduates. Coming from a small liberal-arts college made it easy to lend skulls and pinned butterflies from our collections without fuss or paperwork, and my administration is supportive of local outreach by its faculty. Perhaps the most important factor was that everyone involved had the same goal of guiding children's curiosity about the natural world in ways that would help them become young scientists.

While the elementary school opened its doors to my students, I have not had any luck entering middle or high schools, where the curriculum is prescribed by state-mandated, high-stakes tests, and teachers have much less flexibility. It is a challenge to all of us to see how and where we can join forces with local schools, breaking down barriers between “town and gown.” My metamorphosis from class volunteer to science resource was gradual, and each small step expanded my teaching in ways I could not have predicted. Long after my children graduated from the elementary school, the Science Buddies program continues to link my professional life with hundreds of local schoolchildren. I encourage those who find joy in their laboratories and college classrooms to follow a child into their neighborhood school, sit down in one of those little desks, and prepare to be amazed at what might happen.

**Figure 1:**
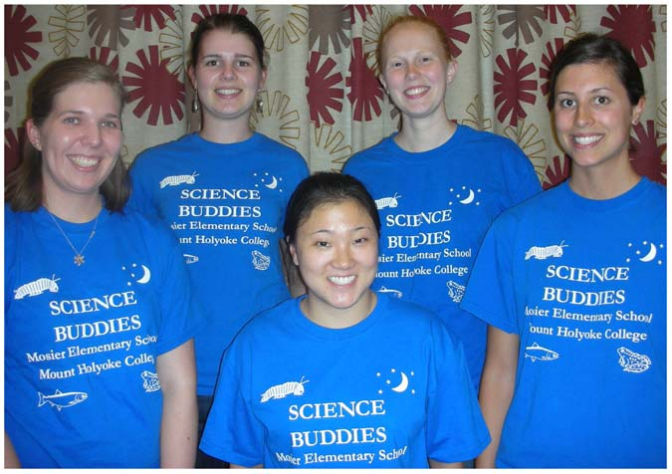
Mount Holyoke College students wearing their Science Buddies T-shirts, at the elementary school Science Fair. (Photo: Rachel Fink).

**Figure 2:**
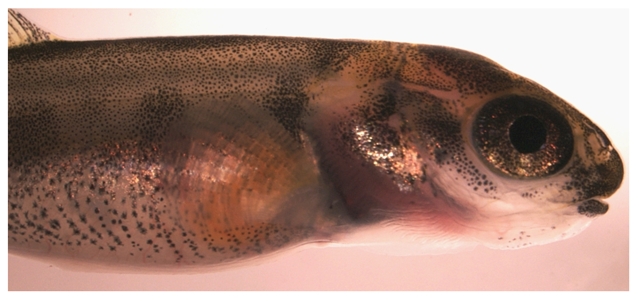
Atlantic salmon larva reared in an elementary classroom as part of a restoration project in Massachusetts. (Photo: Haruka Fujimaki).

